# Remarks on Medical Education

**Published:** 1813-06

**Authors:** 


*To the Editors of the Medical and Physical Journal.*

GENTLEMEN,

IT is often remarked, that few discussions are entirely unattended with some advantage to the public. Such, I trust, will be the case with the present inquiries relative to the *Apothecaries' Bill*; and your Number for April furnishes ample testimony that we may expect important advantages, and such as were little expected by the authors of the undertaking. One writer speaks of ignorant apothecaries, and another seems to undervalue the physician: both, I have no doubt, may be right. But a short letter, signed Juvenis, p. 290, struck me very forcibly, as containing all the difficulties I recollect to have encountered a few years ago. It is too true that too many surgeon-apothecaries take apprentices with no other view than to gain their services in the shop for a certain number of years; and if they sometimes introduce them to a few cases at the workhouse, or among the inferior class of patients, they rarely instruct them how they are to improve by the cases they are allowed to treat. What is still more to be lamented, too many considerable practitioners are actually ignorant of the first principles of their art; for parents, in apprenticing their children, look only, as we might expect, to the quantity of business, and not to the medical knowledge of the master. If the latter is such as I have described, he will soon teach his apprentice to laugh at all theory, as he calls it; but, in doing so, he teaches him also to undervalue all reasoning. The youth thus armed, and, perhaps, as another of your correspondents remarks, (J. W. Empiricis Inimicus,*) with an insufficient education prior to his apprenticeship, "coming to London with a mere pestle-and-mortar knowledge, *and there not sufficiently impressed with the importance of his profession,*" can we wonder that he improves so little during a hasty attendance on the hospitals and the London lectures? This leads me back to your correspondent *Juvenis*, whose modesty cannot be too much admired, in requesting to be directed to such books as may be most likely to serve him preparatory to his appearance as a medical student in London, where he will have little time to read, and have occasion very often to regret that he had read so little, now that he might have applied his reading to so good an account.

It will, perhaps, at once occur, that every young man should begin by reading Cullen; but, alas! even Cullen grows a little out of date: however, I would advise Juvenis to read his First Lines; but always to remember that Cullen in that work follows the order of his Nosology. Now, (says your correspondent M. N., in the same Number, p. 272) "I deny that there is any analogy between tetanus and hydrophobia." A good deal more follows, in which the writer regrets the errors into which the well-deserved celebrity of Dr. Cullen, who classes these diseases together, has led physicians. After this he shows the errors of Darwin; and last of all concludes with a sage aphorism of his own, which has, in my little judgment, no other merit than the Greek etymologies of John Brown may afford it. "Tetanus," says he, "is purely asthenic, while hydrophobia rabiosa appears to be sthenic:"† that is, I suppose, tetanus is weak, and hydrophobia rabiosa is strong. But how can we account for so much strength in a disease of weakness, as to fix the muscles as firm as a statue; that is, so firm that the strongest man, or ten of them, cannot move them? Hitherto I have only shown the want of some book to guide the young practitioner, and probably before now, if Juvenis reads this, he will be waiting with anxious expectation to peruse my catalogue. Now, then, it becomes me to acknowledge that I am not so much older than himself to offer my opinion with so much boldness, but I can tell him the manner in

* P. 276, of your Number for April.

† P. 275, note, in the same Number.

which which I have become reconciled to my profession, and even fancy I am pursuing it with advantage to my patients, and progressive improvement to myself.

About six years ago, feeling at that time the same anxieties as Juvenis, I was much struck with a criticism in the Ed. Med. Journal, on Dr. Adams's "Observations on Morbid Poisons."—"The book," says the reviewer, "is excellent: it displays some of those minutiæ in making observations which characterised that great master, who is so justly admired by all, though venerated perhaps with too profound submission by some of his disciples. Dr. Adams is one of Mr. Hunter's most distinguished followers, and his zeal does honor to the school, and the teacher that inspired it."—These are among the introductory remarks of the reviewer; at the close I was particularly struck with the following passage: "This volume is valuable in another point of view, because it inculcates the habit of analysing diseases, and shows the importance of minute attention in tracing the history and progress of every series of morbid action." The perusal of the above, and of the rest of the critique, gave me hopes, that by the work itself I might at least be instructed how to "*analyse disease;*" and I may add, if it did not answer all my expectations, it at least taught the method of acquiring medical knowledge.

But Juvenis must not stop here, nor perhaps begin here. If his master's library will furnish him with Sydenham and Vansweiten, let him read them both. They contain stores of facts, though mixed with matter which will often tire him; but let him reflect, that if he does not read them now, he will probably never do it hereafter. He should also peruse the Transactions of the London College, and Medical and Philosophical Societies, Collections of Cases, &c. &c. I would wish to encourage him also to continue his contributions, especially as it may teach him the habit of drawing up cases, in doing which he will feel the necessity of minute observation. Before I conclude let me add, that, as the "Morbid Poisons" is an expensive work, I would recommend Juvenis to read with attention the *octavo* edition of Hunter on the Venereal Disease, with Dr. Adams's Commentary, the introduction to which contains a comprehensive sketch of some of the most important doctrines of Mr. Hunter. I am, Gentlemen,

Your's, &c.

EPHEBUS.

